# Negative pressure wound therapy (NPWT) on closed incisions to prevent surgical site infection in high-risk patients in hepatopancreatobiliary surgery: study protocol for a randomized controlled trial—the NP-SSI trial

**DOI:** 10.1186/s13063-020-04831-z

**Published:** 2020-11-09

**Authors:** Frank W. Brennfleck, Lena Linsenmeier, Henrik H.G. Junger, Katharina M. Schmidt, Jens M. Werner, Daniel Woehl, Florian Zeman, Ingrid Mutzbauer, James A. Hutchinson, Edward K. Geissler, Hans J. Schlitt, Stefan M. Brunner

**Affiliations:** 1 Department of Surgery, University Hospital Regensburg, Franz-Josef-Strauß Allee 11, 93053 Regensburg, Germany; 2 Center for Clinical Trials, University Hospital Regensburg, Franz-Josef-Strauß-Allee 11, 93053 Regensburg, Germany

**Keywords:** NP-SSI, SSI, NPWT, ciNPWT, Incision management, Prevena, Wound infection, HPB surgery, Randomized controlled trial

## Abstract

**Background:**

Incisional surgical site infections (iSSI) in hepatopancreatobiliary (HPB) surgery usually lead to prolonged hospital stays, consume valuable resources, and impact on patients’ outcome. Prophylactic closed incision negative pressure wound therapy (ciNPWT) to decrease wound complications has become available. Owing to an increasing number of studies, evidence for superiority in many indication areas has accumulated; however, in general surgery, there are a few data and those have shown contradictory results.

**Methods:**

In this monocentric, prospective, randomized, controlled, two-armed study, the influence of ciNPWT on incisional surgical site infection rates after HPB operations will be investigated. A total of 222 patients will be randomized 1:1 to an interventional group (7-day treatment with ciNPWT) or a control group (treated with gauze dressing). The primary parameter to evaluate efficacy is the rate of incisional SSIs within 30 days after surgery. Additionally, several clinically relevant secondary outcomes will be assessed.

**Discussion:**

A reduction in the rate of incisional SSIs would not only lead to a significant cost reduction and shorter postoperative length of stay, but may also improve postoperative quality of life for patients. While earlier publications have shown advantages for ciNPWT, recent studies did not confirm a positive effect regarding iSSI rate. Even if iSSI rate is not reduced, findings obtained from the secondary endpoints may be of clinical relevance, such as reduction of wound complication rates.

**Trial registration:**

This trial has been registered in the German Clinical Trials Register, DRKS 00015136. Registered on 19 February 2019 and has been approved by the local ethics committee of the University of Regensburg: 18-1225-101.

## Rationale

Surgical site infections (SSIs) are common complications of surgical interventions and occur after 2–5% of all operations across all surgical indications [1, 2]. SSIs account for a large proportion of nosocomial infections [3, 4] and often lead to revision operations [5], prolonged hospital stays, increased costs [6, 7], and a reduction in patient quality of life [8]. In addition, they have been shown to worsen the overall outcome in oncological patients [9, 10].

According to the Center of Disease Control (CDC) classification, SSIs are divided into 3 subgroups. Infections of the skin and subcutaneous tissue form the group of superficial SSIs. If deep structures such as fascia and muscles are affected, they are classified as deep incisional SSIs. Organ space SSIs are indicated when the infection focus is intraabdominal, e.g., in the context of anastomotic leakage [11]. Superficial and deep incisional SSIs together form the group of the incisional surgical site infections (iSSI).

In HPB surgery, which usually involves large open surgical procedures, the described iSSI risk is significantly increased compared to other procedures. They occur at a rate of 8.5–31.5% [12], depending on wound contamination class and the scope of the surgical procedure. A retrospective evaluation of wound infections after HPB surgery in 2015 and 2016 in our department revealed that iSSIs occur after approximately 21% of all open operations; this rate increases to 25% in patients older than 49 years. Therefore, the elucidation of prevention strategies is needed and requires further clinical trialing.

Closed incision negative pressure wound therapy (ciNPWT) has been investigated as a means to prevent wound infections related to certain closed surgical incisions [13]. With this type of approach, the wound is sealed immediately after skin closure under sterile conditions with a special foam-foil bandage, followed by placement of a negative pressure device. Unfortunately, there are only a limited number of randomized controlled trials that have proven the utility of these devices for specific surgeries. In particular, there is little data available showing the value of ciNPWT for the indication of HPB surgery. Therefore, we have designed a prospective randomized clinical study to evaluate whether ciNPWT is a beneficial tool for the prevention of iSSIs in HPB surgery.

## Prior studies

In 2006, Stannard et al. first described the application of negative pressure to primarily closed wounds to prevent wound complications and iSSIs. In their randomized controlled trial (RCT) in patients with high-energy trauma of the lower extremity, they showed a non-significant (*P* > 0.05) reduction in wound infection rates from 16 to 8% [13]. In 2012, a significant reduction of iSSIs from 19 to 9% (*P* = 0.049) in a further RCT in patients with fractures of the lower extremity was found [14]. Indeed, there have been mixed outcomes in other trials. Three randomized trials in orthopedic trauma patients did not show a significant difference in iSSIs [15–17]; however, the AIMS Study did reveal a significant decrease in iSSIs after inguinal vascular surgery; they were impressively decreased in the intervention group to 13.2%, compared to 33.3% in control patients (*P* = 0.0015) [18]. Although information on the principle of action can be derived from these studies, the results are not easily transferable to patients in abdominal surgery due to the changed anatomical and pathophysiological conditions. Therefore, studies on abdominal surgical patients are indispensable.

In the current meta-analysis of randomized controlled trials of ciNPWT from Wells et al., 10 RCTs were identified [19]. Five of these studies investigated the procedure in patients after cesarean section [20–24]. These short operations on usually young healthy adults with small incisions are in no way comparable with the long HPB operations on older, usually significantly sicker patients. In the study of Murphy et al. [25] exclusively and in those of Li et al. [26] and O’Leary et al. [27], almost exclusively colorectal patients were examined. These procedures are also not easily distinguishable with regard to the risk of wound infection and the wound contamination class. While stool contamination of the wound is more frequent in colorectal patients, in HPB surgery this is only done through bile or small intestine stool, but at a significantly lower frequency. On the other hand, perioperative diseases such as blood loss, hypalbuminemia, or liver insufficiency are much more common in HBP patients. Therefore, of the 10 RCTs, only 2 remain in which at least pancreatectomy patients were examined. Shen et al. investigated the influence of ciNPWT on iSSI rate in patients after resection of intraabdominal neoplasia of the stomach, pancreas, and peritoneum; no difference in the frequency of wound infections was observed [28]. However, in this study, 47% of patients underwent CRS/HIPEC surgery, including hyperthermic intraperitoneal chemotherapy following extensive visceral resection, and 21% underwent colorectal surgery. So only 32% pancreatomy patients were included. In addition, negative pressure therapy was applied for only 4 days and a custom-made negative pressure system was applied, which the surgeon had to fabricate himself. Here a performance bias cannot be excluded. In addition, the surgeon was informed of the randomization before the intervention. Only Javed et al. did demonstrate a substantial reduction in iSSI rate after pancreato-duodenectomy using ciNPWT but also only for 4 days (31.1% to 9.7%) [29]. RCTs that examine ciNPWT for liver resections, bile duct surgery, and upper abdominal vessels have not yet been published.

Numerous cohort and retrospective studies have shown effects of ciNPWT, but the evidence level is considerably lower than with the RCTs. In a retrospective analysis of 254 colorectal resections, 32 patients receiving treatment with negative pressure showed a decrease in iSSI rate from 29 to 12.5% (*P* = 0.036) [30]. In another retrospective data analysis where 59 patients after abdominoperineal resections were evaluated, a reduction from 13/32 iSSIs in a control group to 4/27 iSSIs in patients with NPWT (*P* = 0.01) was reported [31]. Pellino et al. and Selvaggi et al. obtained similar results when evaluating patients undergoing colorectal surgery. Selvaggi et al. found a reduction from 12/25 to 2/25 SSIs (*P* = 0.04) in Crohn’s disease patients after bowel resections [32], and Pellino et al. reported a reduction from 11/25 to 2/25 iSSIs (*P* = 0.008) in colorectal surgery patients [33]. In patients receiving surgical abdominal wall repair, two retrospective studies detected a decrease in infection rate from 17/32 to 5/29 (*P* = 0.01) [34] and from 27/84 to 10/115 (*P* = 0.001) [35], while other investigators did not find a difference in infection rates after ventral hernia repair surgery using ciNPWT [36]. Despite a lack of clear evidence and contradictory data, the WHO expert group recommends ciNPWT therapy in high-risk patients in guidelines on the avoidance of iSSIs [37].

Therefore, to clarify the controversy of ciNPWT use especially in HPB surgery, we posed the hypothesis that the use of closed incisional negative pressure bandages can reduce the iSSI rate in this subgroup of patients. In our opinion, another RCT on HPB-patients is needed due to lack of evidence mentioned above.

## Investigational device

The investigational device is the Prevena™ Incision Management System (KCI, San Antonio, Texas). This is a ciNPWT system that supplies negative pressure to closed surgical incisions with the intention to lower the incidence rate of superficial and deep incisional SSIs and also surgical site complications (SSCs). In detail, its intended purpose is the application of a special wound dressing on closed surgical incisions to provide 125-mmHg negative pressure to the wound by a small, battery-driven negative pressure device. The negative pressure may reduce edema, seroma, and hematoma, as well as stabilize wound edges which can be related to the development of iSSIs and SSCs. The Prevena system was selected as one of two commercially available ciNPWT systems at the time of study design because it is easy to use, it has already been investigated in several studies in other indications, and the initiators of the study have already had positive personal experiences with it. The second system available (PICO, Smith & Nephew) also does not consist of a foam dressing and the pump unit does not have an exudate collection tank, which we consider a disadvantage if there is a large outflow from the wound.

Four Prevena™ System types will be used for the study:

1. For linear incisions < 13 cm: PREVENA™ PEEL & PLACE™ System Kit – 13 cm
2. For linear incisions ≥ 13 to < 20 cm: PREVENA™ PEEL & PLACE™ System Kit – 20 cm
3. For linear incisions ≥ 20 to < 35 cm: PREVENA PLUS™ System with PEEL & PLACE™ 35 cm dressing
4. For linear incisions > 35 cm (and linear incisions ≥ 20 to < 35 cm, until PREVENA PLUS™ System with PEEL & PLACE™ 35 cm dressing is available in Europe) or non-linear incisions: PREVENA PLUS™ Customizable System Kit

**Fig. 1:**
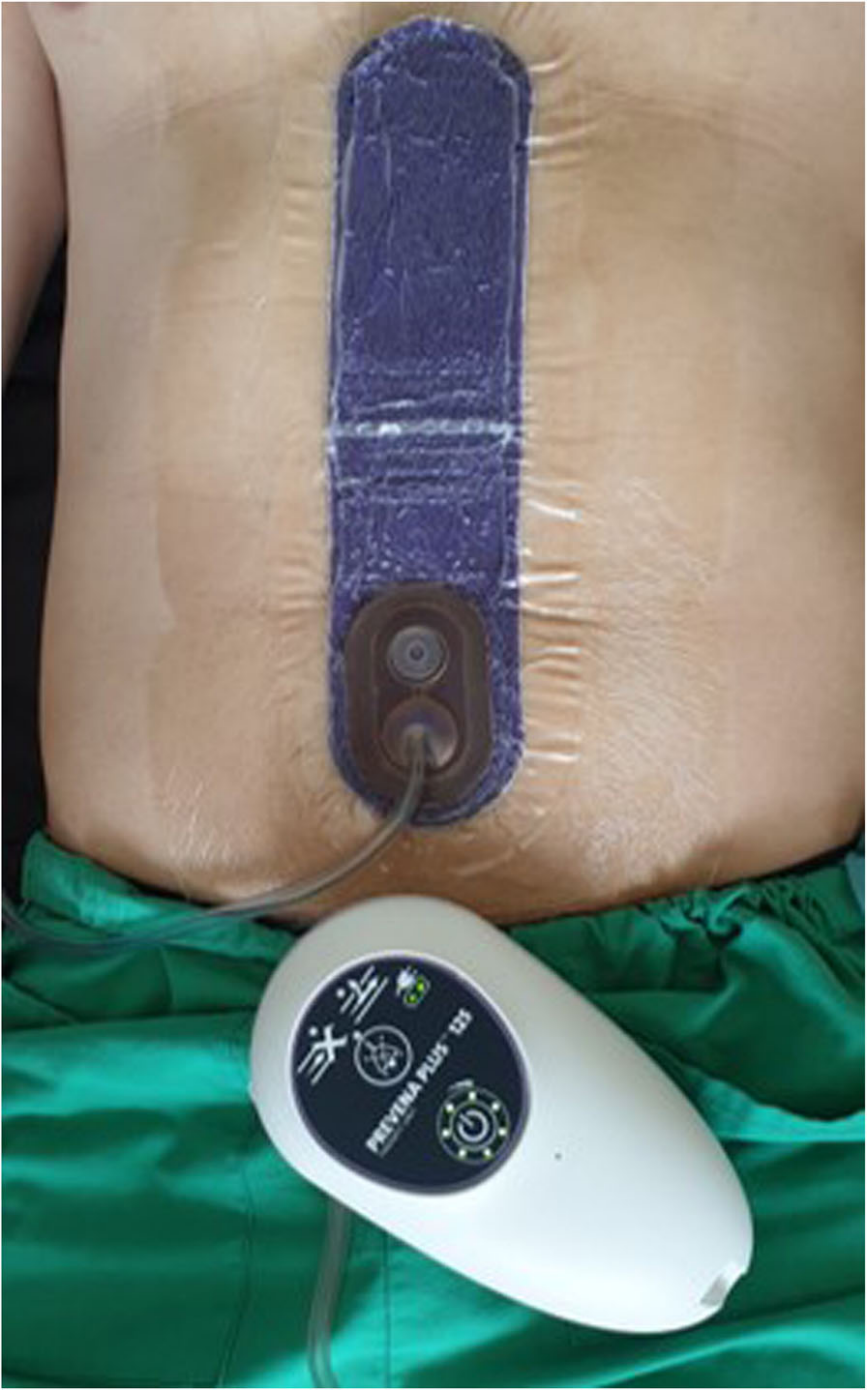
Applied PREVENA™ PEEL & PLACE™ dressing on a patients’ wound, connected to the PREVENA PLUS™ device. The 20-cm long dressing covers the median laparotomy. The Prevena™ Plus device is connected to the dressing via a thin plastic tube and provides a vacuum of − 125 mmHg

Figure 1 shows an example of the applicated PREVENA™ PEEL & PLACE™ 20-cm dressing on a patient’s wound, connected to the PREVENA PLUS™ device.

All participants (surgeons, nurses, study personnel) will be trained in handling the Prevena™ system prior to initiation of the study. The training is carried out in accordance with the manufacturer’s instructions and mainly relates to the handling of the pump, which, however, only has a switch-on button, a connection cable, and an alarm. All participating surgeons and nurses are already familiar with the basic technology of the NPWT through daily use. The application of a wound dressing is also basic surgical knowledge, which can be assumed. After completion of the training, the correct handling of the product is checked by a required demonstration.

### Trial design

This is a prospective, randomized, controlled, two-armed study comparing ciNPWT dressing versus conventional gauze dressings after HPB operation. The primary parameter to evaluate efficacy is the rate of iSSIs within 30 days after surgery. This is a monocentric study performed in the Department of Surgery at the University Hospital Regensburg, Germany, which is a tertiary referral center for HPB surgery.

All patients aged ≥ 50 years who undergo HPB surgery and give informed consent will be allocated to the study.

The study design is based on the ICH-GCP E6(R2) guideline, and on DIN ISO 14155.

### Study team

The operations are performed at the test center by all surgeons of the department. In addition to the two PIs (senior physicians), there are the head physician and 7 senior physicians. The study team that carries out the assessments on days 7 and 30–37 comprises 4 senior physicians and 2 assistant physicians. The study team is supported by a PhD student and a study nurse.

### Objectives

#### Primary endpoint

**Fig. 2:**
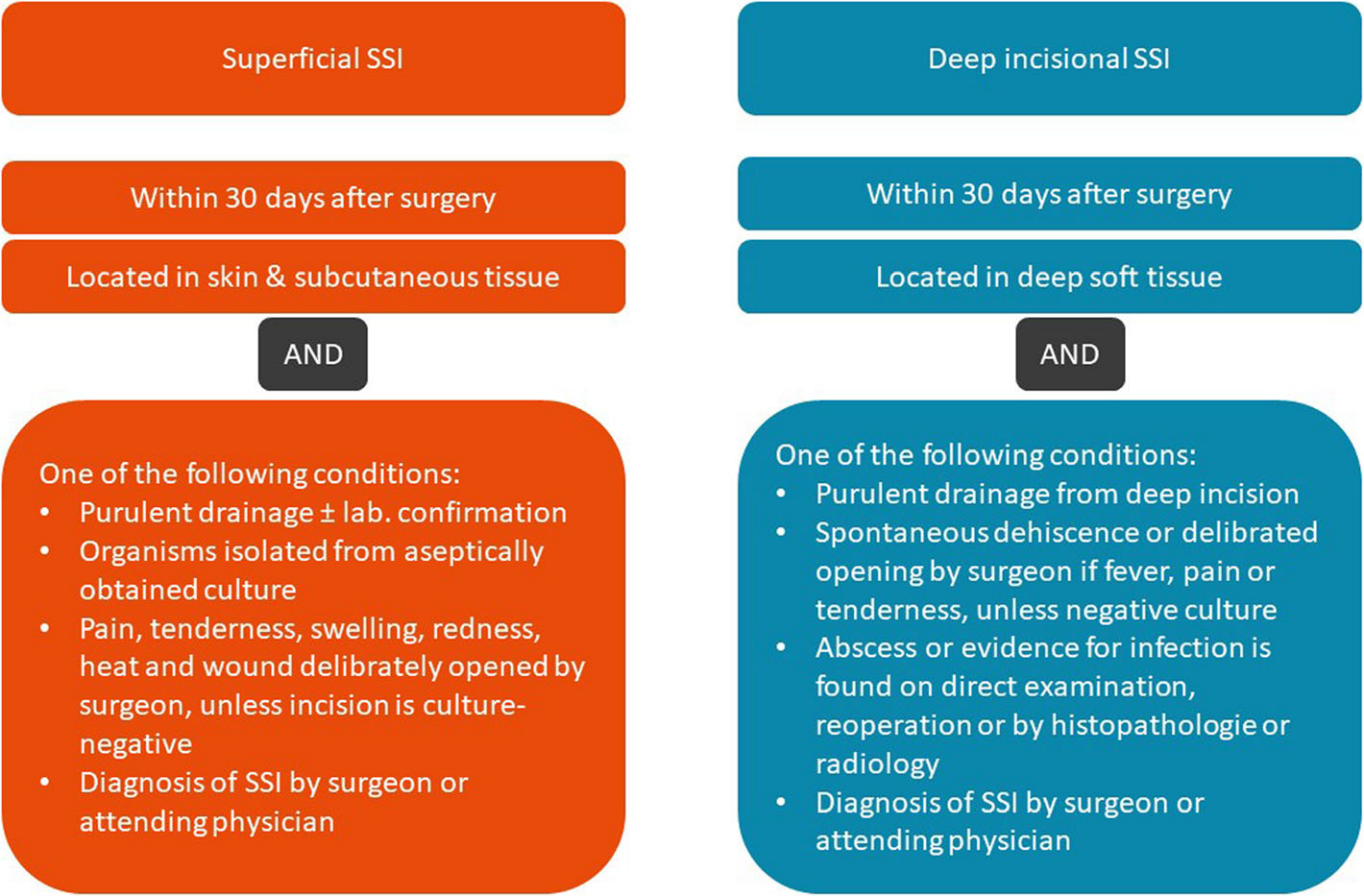
Current CDC-Classification of superficial and deep-incisional Surgical Site Infections. The primary endpoint will be assessed according to the criteria mentioned above

The primary aim of this study is to investigate if ciNPWT provided by the Prevena™ Incision Management System (KCI, San Antonio, Texas) reduces the incidence of superficial and deep incisional surgical site infection within 30 days after HPB surgery, compared to standard of care using a sterile gauze dressing (not including ciNPWT). SSIs are diagnosed and classified according to the current CDC-classification only by attending physicians. The diagnostic criteria are shown in Fig. 2. SSIs are defined as infections in the surgical access that occur within 30 days after surgery. In case of superficial SSIs, it is located in the skin and subcutaneous tissue and in case of deep incisional SSIs in the muscle and fascial layers. It is not always easy to distinguish between the two entities, so that they are initially jointly evaluated as iSSI yes/no in the primary endpoint and can be subclassified in further analyses.

#### Secondary endpoints

- Superficial and deep incisional SSI until day 7 (following CDC classification).
  The occurrence of SSI by the 7th day is diagnosed according to the CDC criteria shown in Fig. 2. The main question here is whether SSI could remain undetected during therapy with NPWT dressings.
- Surgical site complications (hematoma/ seroma/ dehiscence/ necrosis) on day 7 and day 30–37.
- These important wound complications also significantly influence the outcome of the patient and often lead to an opening of the wound.
  A reduction in the occurrence of these complications would also be of utmost clinical importance. During the inpatient stay, these endpoints are recorded by the treating physicians. At visit 4, they are determined by investigators at a personal appointment or by the general practitioner who continues treatment.
- Rate of fascial dehiscence until day 30.
  The endpoint is defined as fascial insufficiency requiring treatment within the first 30 days after surgery and is assessed by the investigator.
- Assessment of Quality of Life using the EQ-5D-5L questionnaire on days 7 and 30–37.
  Participants are asked to complete the EQ-5D-5L Quality-of-Life questionnaire on days 7 and 30–37. The aim is to determine whether ciNPWT has a positive or negative impact on the quality of life.
- Need for antibiotic therapy because of SSI.
  In this endpoint, the administration and indication of antibiotic substances beyond perioperative prophylaxis are assessed.
- Rate of secondary interventions and re-operations.
  All reoperations and interventions such as opening of the wound within the first 30 days are recorded by an investigator.
- Iatrogenic opening of the wound until day 30.
  As above.
- Length of hospital-stay.
  An important economic parameter is the length of hospital stay. It is determined from admission to discharge. A possible resumption within the observation period of 30 days is separately documented and counted.

### Inclusion criteria

The study will include all patients undergoing elective open HPB surgery who are older than 49 years of age and who consent to the study protocol. Based on current WHO recommendation and an international consensus group [37, 38], preventive ciNPWT should only be used in iSSI high-risk patients. In the retrospective analysis of the iSSI in the HPB patients of our department, we identified the group of over 49 year olds with 25% iSSI vs. 13% iSSI among under 49 as a high-risk group and therefore selected them as the study population.

### Exclusion criteria

The following patients will be excluded: (1) those not meeting the inclusion criteria; (2) patients with social, geographic, or family conditions that compromise compliance with the study protocol; (3) those cases where primary wound closure is not achieved; and (4) patients undergoing a planned second-look laparotomy.

### Interventions

#### Randomization

After the skin is closed during surgery, the investigator opens the closed randomization envelope with the lowest numbering containing the unique patient identification number and allocation designation to one of the following treatment groups. These block randomized envelopes will be prepared and closed by the external study-statistician (center for clinical trials, University Hospital Regensburg). The randomization is not stratified. In order to make the conduct of the study as simple as possible, we have chosen non-stratified randomization. In principle, stratification would have been possible and useful to homogenize the distribution of high-risk patients within the groups.

#### Intervention group

The wound is bandaged in the operating room under sterile conditions with a ciNPWT dressing of appropriate size and configuration (longitudinal, transverse or L-shaped). The Prevena™ device contained in the dressing set is then connected and switched on; in this way, 125-mmHG negative pressure is applied to the wound for 7 days. After 7 days, the device switches off automatically. Due to the non-transparent design of the dressing, the wound cannot be examined during the treatment period. If the attending surgeon strongly suspects an incisional SSI below the ciNPWT dressing because of fever (without other reason), pain, or suspicious laboratory results, the dressing will be removed immediately. If a superficial or deep incisional SSI is diagnosed, the primary outcome has been reached and the incision can be treated independently from the protocol according to our local standard. If no infection is found, ciNPWT will continue for the remainder of the 7-day treatment period by applying a new PREVENA™ dressing [reusing the initial PREVENA™ (or PREVENA PLUS™) 125 Therapy-unit]. Detailed reasons for the therapy interruption will be documented in the electronic case report form (eCRF). Bandage removal without strong clinical indication because of suspicion of SSI will be registered as a protocol deviation.

#### Control group

The wound is bandaged in the operating room under sterile conditions with a gauze dressing (Cutiplast® Sterile, Smith & Nephew) of appropriate size and configuration (longitudinal, transverse or L-shaped); in addition, the type of dressing is documented in the eCRF so that any deviations can be traced. Aseptic dressing changes will be performed as part of the clinical ward routine, as required. If an iSSI is diagnosed during one of these dressing changes, the primary outcome has been reached and the incision can be treated independently from the protocol according to normal local standard. In principle, the more frequent dressing changes could allow more iSSI to be diagnosed in the control group. However, we try to avoid this bias by strictly applying the CDC criteria and performing wound swabs in suspected cases.

#### Re-operations

Depending on the reason and time of a revision operation, the protocol is adapted accordingly. If a patient needs to be revised due to iSSI, the study therapy in both groups is terminated when the dressing is removed and the patient is treated according to our local standards for wound infections (e.g., open wound treatment, negative pressure wound treatment). If a patient is revised for any other reason within the first 48 h after the first operation (e.g., anastomotic insufficiency, bleeding), the ciNPWT dressing or gauze bandage is removed in the operating theater before beginning surgery. A new dressing is applied, depending on the group, after the skin has been closed again. The therapy is restarted at d0. In the case of a revision later than 48 h after the first operation, the associated removal of the dressings is regarded as a protocol violation and the wound is bandaged with a gauze dressing.

#### Implementation of adherence to the intervention protocols

The LOT numbers of the dressings and negative pressure devices used are documented and the respective packaging is archived together with the study documents. The random envelopes are also provided with the patient data and archived. The investigators’ entries are controlled by the PI and verified according to GCP. The monitors carry out the source data verification. The attending physicians are also regularly trained by the study team and receive follow-up training in case of protocol violations or ambiguities.

## Sample size

Sample size is determined according to the primary endpoint and the hypotheses formulated below. The initial sample size was calculated according to the literature and clinical experience (see calculation below). Additionally, a blinded sample size recalculation [39] will be performed after 50% of the initial calculated patients are included. This ensures a far more accurate estimation of the expected response rates and thus a more reliable recalculated sample size (*n*_recalc_). However, more than a total of *n*_max_ = 450 patients is not feasible for this trial and 450 was set as the upper sample size limit. Thus, a total of *n*_recalc_ patients will be randomized to this trial if *n*_*r*ecalc_ ≤ 450. If the *n*_recalc_ is > 450, the study will be terminated.

### Initial sample size calculation

In a retrospective analysis of the iSSI frequency during the years 2015 and 2016 in surgically treated HPB patients from our department, more than 600 patient datasets were evaluated. In the group matching the inclusion criteria of the present study (342 patients), the incidence of iSSI was approximately 25%, including 15% superficial and 10% deep-incisional SSIs. All of these iSSIs led to re-opening of the wound with consecutive secondary-intended healing or an additional operation.

In the present trial, we expect to achieve a reduction in incisional SSI rate from 25 to 10% or less, which would be an absolute reduction rate of 15% and a relative reduction of approximately 60%. In the AIMS Study, Gombert et al. [18] showed a relative risk reduction of 60% in vascular surgery patients following groin incisions. Javed et al. [29] demonstrated a reduction of 69%. In the RCTs from other indication areas, in which a positive effect of ciNPWT was shown, risk reduction rates of 60% were also shown [26, 27]. These data led to our estimation; however, in other studies, only lower risk reduction rates or partially no effect could be shown [28, 36].

To detect a difference of 25% versus 10%, with a power of 1 − *β* = 80%, at a two-sided significance level *α* = 0.05 using a chi-square test of independence without continuity correction and 10% missing items, a total of *n* = 222 patients should be enrolled into the trial. Sample size was calculated using IBM SPSS Sample Power 3 (see below).

This estimate is based on a very optimistic assessment of the expected effect. A much more conservative estimate would have been discussed at this point, although at the time of the study design not all RCTs published today were available. Therefore, the interim analysis presented below was incorporated into the protocol.

### Blinded sample size recalculation

After 50% of the patients (*n* = 111) have reached the primary endpoint (information rate of 0.50), a blinded sample size recalculation will be performed. Preserving blindness is in line with the ICH E9 Guideline on “Statistical Principals for Clinical Trials” and prevents an inflation of the type I error rate. Sample size recalculation will be performed as described in the publication of Friede and Kieser [39]. If more than 450 patients in total will be needed for trial completion, the study effect will be considered as too small and the trial will be stopped due to futility. If less than 111 patients are recalculated to give the required power to detect a significant effect, patient recruitment will be stopped, and the study will be terminated and analyzed after all included patients finished the final visit. Should the interim analysis show that more than 222, but fewer than 450, patients need to be examined, the study should be continued aiming for the indicated increased number of patients.

### Recruitment

**Fig. 3:**
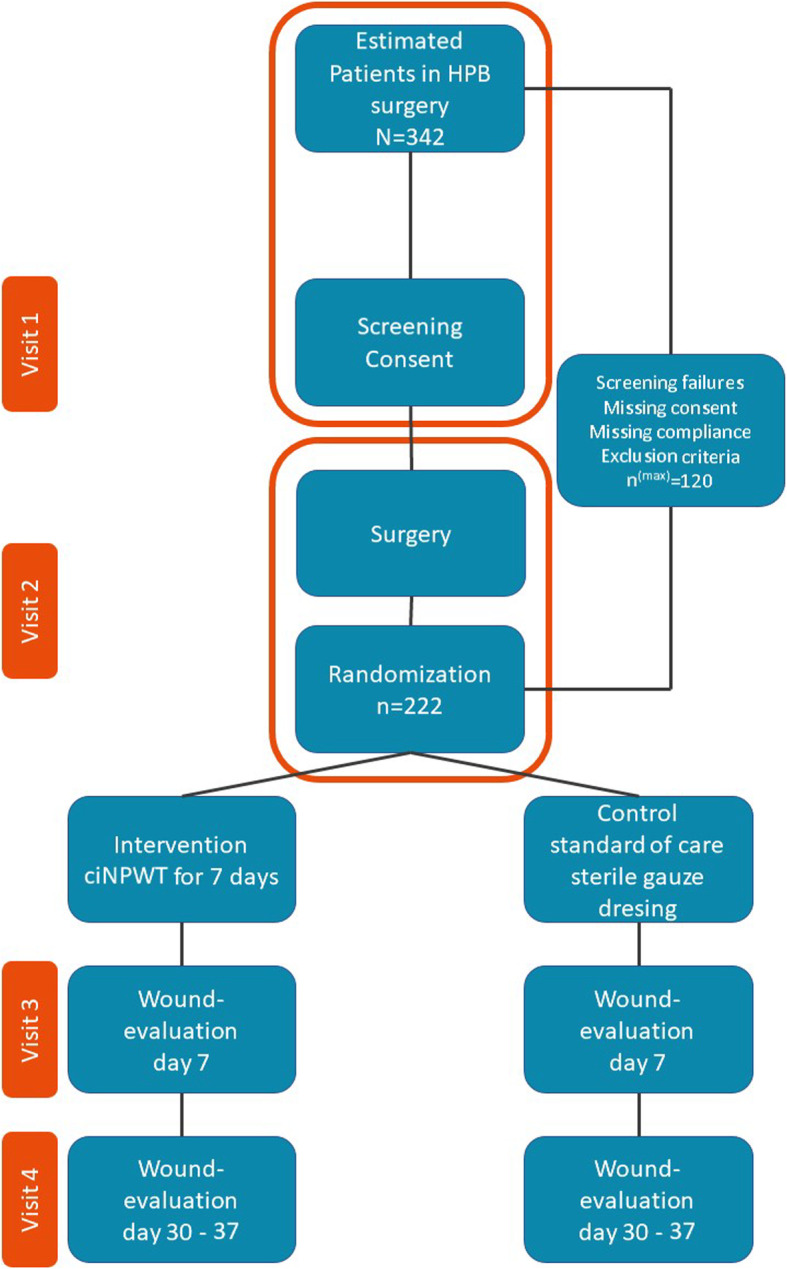
Study timeline. Patients who meet the inclusion criteria will be asked for their consent after information. At the end of the operation, the participants are randomized, and the intervention is performed according to the treatment group. During visit 3 the bandages are removed on day 7 and the endpoints are recorded as on days 30–37

In 2015 and the first half of 2016, 342 patients matching the inclusion criteria were operated on in our department. With an expected participation rate of 66%, about 12 to 13 patients can be included per month. Thus, the initial sample size of 222 patients can be reached after 18 months. The maximum recruitment time with *n*_max_ = 450 patients is approximately 36 months. The study timeline is shown in Fig. 3.

## Data collection methods

### Screening

Planned HPB operations in our surgery department will be screened weekly for potential patients fulfilling the inclusion and exclusion criteria.

### Visit 1 (minimum 1 day before planned operation)

After supplying patient information and obtaining informed consent for the operation procedure, qualified patients will be informed about the NP-SSI trial and receive the patient information sheet; after sufficient time for reflection, patients are asked for their consent to participate in the study. Once consent is obtained, a unique study participant number for the patient will be assigned.

### Visit 2 (operating room; minimum the following day after inclusion)

When the skin is closed after surgery, a randomization envelope with the respective patient identification number will be opened by the investigator and a sterile gauze dressing (control group) or the size-appropriate PREVENA™ dressing will be applied to the wound; this is considered visit 2. Care will always be taken to keep materials for both dressings in the operating room. All investigators will be educated in handling the PREVENA™ dressing and the PREVENA™ device. Additional data which will be documented includes the time from incision to suture (documented in the operation report) and the incision length and configuration. If the wound cannot be closed during the primary operation due to secondary intended healing, or if a second look is planned, the patient will be counted as a screening failure.

During the 7 days of ciNPWT, patients in the negative pressure test group will receive the same medical care and supportive therapy as the control group. Nevertheless, dressing changes and wound controls are performed only in the control group, since these bandages are changed often and allow inspection of the wound. If an iSSI is strongly suspected in the intervention group by the attending surgeon due to fever or other symptoms, the dressing will be removed by the surgeon using aseptic technique and the iSSI will be treated if there is visible evidence. If no iSSI is diagnosed, the ciNPWT therapy will be continued using a new dressing to complete the 7-day treatment period by applying a new Prevena dressing under sterile technique, but the same PREVENA™ (or PREVENA PLUS™) 125 Therapy unit will be reused. This will be documented in the CRF.

### Visit 3 (7 days after visit 2)

Dressings in the intervention group will be removed and the wound assessed by an investigator. Control group dressings will be changed over the course of 7 days if necessary and wounds examined during each dressing change. On day 7, record will be made of an iSSI occurring anytime over the 7 days and in those cases, the date of diagnosis, type of iSSI (superficial, deep incisional) and note if the wound had been opened post-surgically. Furthermore, we will record if the ciNPWT therapy had been interrupted, if a second dressing had been applied and the duration that ciNPWT was applied. In control group patients, the number of dressing changes will be logged. Additionally, the use of antibiotics and the specific reason for usage will be documented (e.g., was it used to treat a surgical wound infection?). Wounds also will be examined with respect to SSC (hematoma, seroma, dehiscence, necrosis). Any re-operation or re-intervention will also be documented. At this time point, patients will be asked to complete the EQ-5D-5L questionnaire.

### Visit 4 (days 30 to 37 after visit 2)

Ideally, patients will be present in the clinic during our routine surgical follow-up on days 30 to 37. Wounds will be examined by the attending surgeon and the occurrence of iSSI or SSC in the intervening period after post-operative day 7 will be documented. If the patient is not able to be present at the study site, endpoints will be assessed by a standardized interview and the EQ-5D-5L will be completed via a telephone call or by an online questionnaire sent to the patient by the data management software. Patients will also be asked to upload a surgical wound photo to remotely confirm the information given by phone interviewing. The interview or online survey will be performed between days 30 and 37 after visit 2. If the answers in the telephone interview are unclear and there is an indication for an iSSI or SSC within the 30 days after operation, the study team will contact the actual treating physician, family practitioner, or the rehabilitation practitioner to get further information (after the patient has given consent). The following parameters will be evaluated during visit 4: (1) iSSI within 30 days after operation, (2) iatrogenic wound opening, (3) SSC on day of visit 4 (hematoma, seroma, dehiscence, necrosis), (4) occurrence of a re-operation or re-intervention, (5) the use of antibiotics within 30 days after operation, and (6) the rate of fascial dehiscence (Table 1). If the patient develops an iSSI during the hospitalization period earlier than visit 4, and if this is confirmed and documented by the attending surgeon, the primary endpoint is reached. In addition, the length of hospital stay will be recorded at visit 4.

**Table 1:**
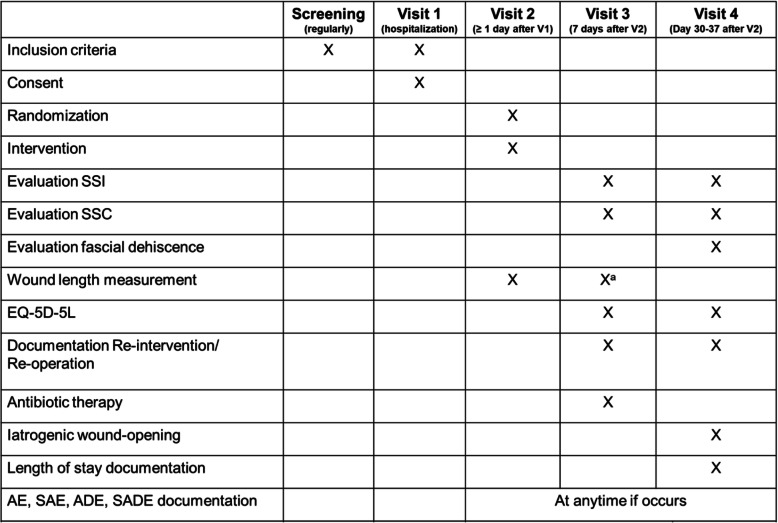
Timeline of procedures and data collection—NP-SSI-trial

^a^if not documented during visit 2

AEs and SAEs will be assessed at visits 2–4, and otherwise when they occur.

## Statistical methods

### Objectives

The primary objective of this study is the assessment of efficacy of Prevena™ dressings in the treatment of closed surgical incisions after an HPB operation. Efficacy is evaluated by the occurrence of a surgical site infection.

### Hypotheses

The study objective can be formulated as a test of the null hypothesis H0: πi = πc versus the alternative hypothesis H1: πi ≠ πc, where πi and πc represent the relative frequencies of SSI in the intervention and control group.

### Population for analysis

The primary analysis will be based on the intention-to-treat (ITT) analysis set. However, a sensitivity analysis will be done on a per-protocol (PP) analysis set. The latter serves to assess the robustness of the results. All safety data will be analyzed by means of the safety population.

#### ITT analysis set

This consists of all patients who entered the study (i.e., all patients who received a patient identification number). According to the IIT principle, all patients will be analyzed as belonging to their randomized device, regardless of whether the device was refused or removed, or whether other protocol deviations are known.

#### PP analysis set

This consists of the ITT analysis set with no major protocol violations. Major and minor protocol deviations will be identified by medically trained staff before database lock.

#### Safety population

This consists of all patients who received one device and who had at least one post-baseline safety assessment. The statement that a patient had no adverse events also constitutes a safety assessment.

### Statistical analysis

The statistical analysis will be done by the Center for Clinical Studies at the University Hospital Regensburg. Statistical analyses will be carried out using SAS 9.4 or higher.

Unless otherwise specified, all statistical tests will be two-sided and will be done at the 0.05 significance level. Because all secondary endpoints are of exploratory character, no adjustment for multiple testing will be performed. Thus, except for the primary endpoints, the results are not confirmative.

#### Patient demographics and other baseline characteristics

Demographical and other baseline data (including disease characteristics) will be summarized for all patients. Categorical variables (e.g., gender) will be presented as the number and percentage of patients in each category. Continuous variables (e.g., age) will be summarized by means of descriptive statistics (*N*, mean, standard deviation, median, interquartile range [25th to 75th percentile], minimum, and maximum). The documented variables are listed in Table 2.

**Table 2:** Demographics and baseline characteristics

Demographics/baseline characteristics	Quality
Patient age	Numeric
Diagnosis	Nominal
Gender	Nominal
Length	Numeric
Height	Numeric
BMI	Numeric
Diabetes	Nominal
Insulin medication	Nominal
Immunosuppression	Nominal
Prior Laparotomy	Nominal
Malignancy	Nominal
Ex-smoker	Nominal
Active smoker	Nominal
Steroid medication	Nominal
COPD	Nominal
Coronary artery disease	Nominal
Arterial hypertension	Nominal
ASA Classification	Ordinal

#### Investigational devices

Descriptive statistics characterizing use of the investigational devices will be provided for both treatment arms.

#### Primary endpoint

The presence or absence (y/n) of an iSSI within the first 30 days after operation is defined as the primary endpoint. Absolute and relative frequencies with corresponding 95% confidence intervals will be presented as point estimates for both groups. The rate of iSSIs will be compared between both groups by using a chi-square test of independence. The results will be presented in a contingency table and the odds ratio as well as the relative risk, both accompanied by the corresponding 95% confidence interval, will be presented as effect estimates. All efficacy analyses will be performed on the ITT population and will be two-sided at the significance level of 0.05. All statistical analyses will be performed using SAS version 9.4 or higher.

#### Secondary endpoints

All secondary endpoints will be analyzed in a purely exploratory manner. Thus, *p* values and corresponding confidence intervals are only descriptive in nature.

All secondary endpoints will be summarized using descriptive statistics. Categorical data will be expressed as frequency counts and percentages. Continuous variables will be summarized by means of descriptive statistics (*N*, mean, standard deviation, median, interquartile range [25th to 75th percentile], minimum, and maximum).

#### Safety endpoints

All safety data will be listed. Adverse events will be presented in frequency tables. In addition, adverse events will be tabulated by severity and relationship to study device.

### Interim analysis

A blinded sample size recalculation will be performed after inclusion of half of the initial calculated patients (information rate 0.5). Possible consequences are termination of the trial early due to futility, efficacy, or adaptation of the sample size as described above.

### Missing data

No imputation methods on missing values regarding the primary endpoint will be used for the primary efficacy analysis. Nevertheless, results will be validated by imputing missing information about the primary endpoint SSI (y/n) conservatively as having an iSSI.

### Cross over therapy/non compliance

Cross over therapy is not to be expected as the study treatment only takes place once in the operating theater. In principle, non-compliance is possible to the extent that the study participant could manipulate the dressing or pump (switch it off, disconnect it). This would be documented as a protocol violation.

### Monitoring

Monitoring of the trial data will be performed by coTrial Associates (www.cotrialassociates.com), which is located within our Department of Surgery. Monitoring will be carried out in accordance with standard operating procedures of the coTrial Associates, using a risk-based approach. Regular on-site monitoring visits will be performed. Investigators must allow the monitor to look at all source data and essential documents, support the monitor during visits and answer queries. All monitoring procedures and the extent of Source Data Verification (SDV) will be predefined in a trial-specific monitoring manual.

### Safety

There is no additional risk expected for patients treated with ciNPWT. Safety and effectiveness have been shown in other indications in several studies before [17, 18, 29–31, 36, 40]. The PREVENA™ device is CE certified and is approved for this indication.

All adverse events and effects will be documented in the eCRF and will be classified as serious and non-serious, expected or unexpected and study-related, possibly study-related, or not study-related by the investigator and reported to the sponsor (University Hospital Regensburg). Adverse events (AE) and adverse device effects (ADE) need to be reported as soon as possible, but within 1 week after becoming known at the latest; serious adverse events (SAE) and serious adverse device effects (SADE) must be reported immediately to the sponsor within 24 h after becoming known. Because of the non-interventional character of the study regulated by §23b of the German law on medical products (MPG), there is no need to report SAEs to the German national authorities. SAEs and SADEs are reported regularly to Kinetic Concepts Inc. (KCI, San Antonio, Texas) using the following email address: Complainthandling@acelity.com. The report should contain date of adverse event, treatment, resolution, seriousness, and the relationship to the investigational device.

## Discussion

Incisional SSIs account for a major part of the hospital-acquired infections in Germany [41]. Particularly after major open operations, such as HPB surgery, iSSIs occur at a frequency of 8–31.5%, depending on type of operation and wound contamination class [12, 42]. In a retrospective analysis of open-surgical operations in HPB surgery in 2015 and 2016 in our department, we found that 22% of all patients developed an iSSI. Patients older than 49 years were at an even higher risk, developing iSSI in 25% of all cases. These iSSI rates are comparable with those in the current literature, although the information varies considerably depending on the publication. Nakahira et al. found iSSI rates of about 22% in HPB patients [42], while Molena et al. only found iSSI rates of 4.73% after hepatic resections and 2.75% after colectomies evaluating data from the American College of Surgeons National Surgical Quality Improvement Program from 2005 to 2012. This study however included interventions of various degrees of resection and was comprised of both open and laparoscopic surgeries [5]. Each iSSI leads to a substantial increase in costs for health care providers. In more detail, depending on the department specialization and iSSI severity, these complications account for additional costs between $4000 and $40,000$ per case [43]. But not only economic aspects should be considered. Wound infections represent a great burden for the patient that leads to quality-of-life loss [8]. Moreover, a direct influence on the overall outcome for the patient has been demonstrated. Buettner et al. showed a deterioration of recurrence-free survival and overall survival after curative-intended resections of extrahepatic bile duct malignancies [9]. Furthermore, similar results have been shown in cases of hepatic resection of colorectal liver metastases [10]. It has also been shown that important therapies such as adjuvant chemotherapy begin at delayed course due to iSSI occurrence [44]. Because of these and other factors, research aimed at reducing the frequency of incisional SSIs is expected to be beneficial both for the patient and health care systems.

Within the scope of this study, however, negative pressure therapy will not only be investigated regarding the infection rate. The secondary endpoints we have chosen also are of high clinical relevance. Wound complications, for example, also belong to an entity of postoperative complications whose potential to worsen outcomes should not be underestimated. Seroma and hematoma often lead to an opening of the wound with subsequent secondary healing or promote the development of iSSI. Another considered endpoint is fascial dehiscence, which has various causes, most of which are not necessarily influenced by preventive negative pressure therapy. However, if fascial dehiscence is caused by a descending superficial SSI or deep incisional SSI, rates of dehiscence might be lowered by successful negative pressure therapy. If wound healing conditions are improved with negative pressure therapy, it will likely be possible to reduce HPB surgical complications and thus the length of patient stay, which is of clinical-economic relevance.

There are a number of factors associated with ciNPWT that may help to avoid the adverse events discussed above, while ciNPWT is thought to reduce shear forces at the approximated wound edges [45, 46]; increase blood flow, the capillary venous oxygen saturation, blood flow velocity, and relative amount of hemoglobin [47]; and decrease tissue edema, as already described for conventional NPWT [48]. First described by Stannard et al. in 2006, several commercial closed incisional negative pressure dressing systems have been launched and promoted in recent years [13]. In addition, the number of publications on this topic has steadily increased. In vascular, trauma, and plastic surgery, this method is increasingly established as a preventive treatment for high-risk wounds [14, 18, 49]. However, high level evidence for an effect in abdominal surgery remains elusive, and existing data is contradictory. For instance, the recently published NEPTUN study showed no difference in iSSI rate and length-of-stay in colorectal surgery [25], but O’Leary et al. have shown a significant reduction in iSSI rate from 32 to 8.3% (*P* = 0.043) in an RCT of colorectal, gynecological, and few HPB patients [27]. Despite compelling evidence, ciNPWT is considered in the current guideline for prevention of iSSIs [37]. However, in these times of increasing economic pressure to health care systems, existing clinical trial data does not convincingly justify use of this comparatively cost-intensive technology. Therefore, more randomized controlled trials are needed to determine if ciNPWT is actually useful and economically beneficial, especially in HPB surgery because we have no randomized controlled data on patients following hepatic or biliary resections. We expect that the data and findings obtained with our NP-SSI trial will substantially contribute to this determination.

## Trial status

The currently valid version of the protocol is V-03-2020-1. The recruitment phase started on April 10 2019 until estimated October 2020.

## Data Availability

The datasets analyzed during the current study are available from the corresponding author on reasonable request.

## Abbreviations

ADE: Adverse device effect
AE: Adverse event
BfArM: Bundesamt für Arzneimittel und Medizinprodukte
CDC: Center of Disease Control
ciNPWT: Closed incision negative pressure wound therapy
CRF: Case record file
HAI: Hospital-acquired infections
HPB: Hepatopancreatobiliary
iSSI: Incisional surgical site infection
ITT: Intention to treat
KCI: Kinetics Concepts Inc.
NPWT: Negative pressure wound therapy
OR: Operating room
PP: Per protocol
RCT: Randomized controlled trial
SADE: Serious adverse device effect
SAE: Serious adverse event
SDV: Source data verification
SSC: Surgical site complication
SSI: Surgical site infection
